# Dietary Differentiation and the Evolution of Population Genetic Structure in a Highly Mobile Carnivore

**DOI:** 10.1371/journal.pone.0039341

**Published:** 2012-06-29

**Authors:** Małgorzata Pilot, Włodzimierz Jędrzejewski, Vadim E. Sidorovich, Wolfram Meier-Augenstein, A. Rus Hoelzel

**Affiliations:** 1 School of Biological and Biomedical Sciences, Durham University, Durham, United Kingdom; 2 Museum and Institute of Zoology, Polish Academy of Sciences, Warszawa, Poland; 3 Mammal Research Institute, Polish Academy of Sciences, Białowieża, Poland; 4 Instituto Venezolano de Investigaciones Cientificas (IVIC), Centro de Ecologia, Caracas, Venezuela; 5 Institute of Zoology, National Academy of Sciences of Belarus, Minsk, Belarus; 6 Stable Isotope Laboratory, James Hutton Institute, Invergowrie, Dundee, United Kingdom; Smithsonian Institution National Zoological Park, United States of America

## Abstract

Recent studies on highly mobile carnivores revealed cryptic population genetic structures correlated to transitions in habitat types and prey species composition. This led to the hypothesis that natal-habitat-biased dispersal may be responsible for generating population genetic structure. However, direct evidence for the concordant ecological and genetic differentiation between populations of highly mobile mammals is rare. To address this we analyzed stable isotope profiles (*δ* ^13^C and *δ* ^15^N values) for Eastern European wolves (*Canis lupus*) as a quantifiable proxy measure of diet for individuals that had been genotyped in an earlier study (showing cryptic genetic structure), to provide a quantitative assessment of the relationship between individual foraging behavior and genotype. We found a significant correlation between genetic distances and dietary differentiation (explaining 46% of the variation) in both the marginal test and crucially, when geographic distance was accounted for as a co-variable. These results, interpreted in the context of other possible mechanisms such as allopatry and isolation by distance, reinforce earlier studies suggesting that diet and associated habitat choice are influencing the structuring of populations in highly mobile carnivores.

## Introduction

Recent studies on highly mobile carnivoran mammals have revealed cryptic population genetic structures that correlate to transitions in habitat types and prey species composition [1]–[10]. For example, in the Canadian lynx *Lynx canadensis*, three genetically distinct units were revealed throughout the species range, which corresponded to three demographically distinct populations, each with synchronized population cycles, inhabiting three climatic regions [1]. Further study suggested that differential snow conditions between these regions leads to different predator-prey dynamics of the lynx and its main prey, the snowshoe hare *Lepus americanus*, which de-synchronizes population cycles between regions and leads to the genetic structure [2]. Other examples show genetic differentiation that appears to be directly correlated to prey specialization. Dalén *et al*. [4] demonstrated that the genetic structure of the Arctic fox *Alopex lagopus* throughout its range corresponds to two ecotypes, one specialized on lemmings and another feeding on coastal food. Habitat and dietary differentiation has also been shown to correlate with genetic structure in the grey wolf *Canis lupus* [7]–[10]. More similar examples can be found in some social Odontocete (toothed whale) species that also show cryptic population subdivisions correlating with habitat structure or prey specializations (see review in [11]). For the killer whale *Orcinus orca*, it has been proposed that social learning about how and where to exploit reliable prey resources promotes philopatry, and leads to the genetic differentiation between sympatric and parapatric populations that specialize on different prey resources [12].

It could be argued that differentiation with respect to diet may be expected among populations that have been isolated enough to differentiate genetically, and that there may be no causative relationship. However, it is not always the case that resource specialization is correlated to population genetic structure. For example, sympatric habitat specialist oystercatchers (*Haematopus ostralegus*) showed no sign of genetic differentiation (in contrast to sympatric killer whale specialists, see above) [13], and the pattern of speciation among sibling species of triplefin fish (Tripterygiidae) did not always follow expectations based on resource specializations [14]. There is a need to define the specific mechanisms, in order to better understand why reproductive isolation is promoted in some cases and not others.

A candidate mechanism leading to genetic differentiation based on resource specialization is biased dispersal towards familiar resource or habitat. Non-random dispersal, possibly directed by suitable habitat or resource acquisition, may lead to genetic structuring and evolutionary differentiation by genetic drift. For example, a study of a local population of great tits *Parus major* showed that non-random dispersal driven by differences in habitat quality may reinforce rapid evolutionary differentiation over spatial scales that are surprisingly small relative to the dispersal range of the species [15]. Behavioral studies on a broad range of animal taxa indicate that dispersing animals exhibit preferences toward habitats containing cues comparable to those in their natal habitat [16]. However, most evidence gathered so far that habitat and/or dietary differentiation may promote genetic differentiation at neutral markers has been indirect, based on correlative studies of populations living in different ecological conditions. More direct evidence is rare, however a study that combined direct tracking of individual coyotes (*Canis latrans*) with the analysis of genetic differentiation demonstrated that conservative habitat selection among individual dispersers served as a proximate mechanism for genetic structuring [5].

In this study, we analyzed the diet (as reflected in stable isotope profiles) of individual European grey wolves with known genotypes in an area where cryptic population genetic structure has been earlier revealed. Pilot *et al.* [7] studied 643 wolves from 59 locations representing most of this species distribution in eastern Europe and found population genetic structure that correlated with climate, habitat type and diet composition. In that study, the correlation between prey choice and population structure was studied using the mean share of four ungulate prey species in wolf diet (based on published data about the frequency of a given species among the ungulates killed by wolves in a given area). However, the data on wolf diet were only available for 17 out of 59 regions studied, and in most cases they were not collected in exactly the same locality as the samples for the genetic analyses. For regions where prey composition could not be included, another environmental variable tested, the vegetation type, was the most important variable in terms of the strength of the correlation and the percentage of genetic variation explained [7].

Where diet composition could be tested, the strength of the correlation depended on the genetic markers used (mitochondrial vs nuclear). However, because the species composition of ungulates (being the staple prey of wolves) strongly depends on habitat type and climate, genetically differentiated wolves may be expected to differ in diet composition, either due to differential prey choice, or another habitat cue correlating with prey composition. We therefore use stable isotope analysis to obtain discrete information on the individual diet of wolves for which microsatellite and mtDNA genotypes have been obtained in the earlier study [7]. We test the hypothesis that dietary data explain the greatest proportion of neutral genetic variance for local populations of the grey wolf in comparison with data on geographic distance – a result expected if wolf population differentiation is shaped by ecological factors rather than geographical constraints.

## Materials and Methods

### Material

We analyzed stable isotope profiles (expressed as *δ* ^13^C and *δ* ^15^N values) of muscle tissues of 110 grey wolves from Russia, Belarus, Latvia, Ukraine, and Poland. They represented a subsample of 317 individuals from 15 geographical regions that were analyzed for mitochondrial DNA (mtDNA) control region and 14 microsatellite loci by Pilot *et al.* [7]. That earlier study had found support for two cryptic subpopulations as defined by the microsatellite DNA data (here referred to as NUC1 and NUC2), and further subdivision based on mtDNA data (MIT1 – MIT4) [7].

**Figure 1:**
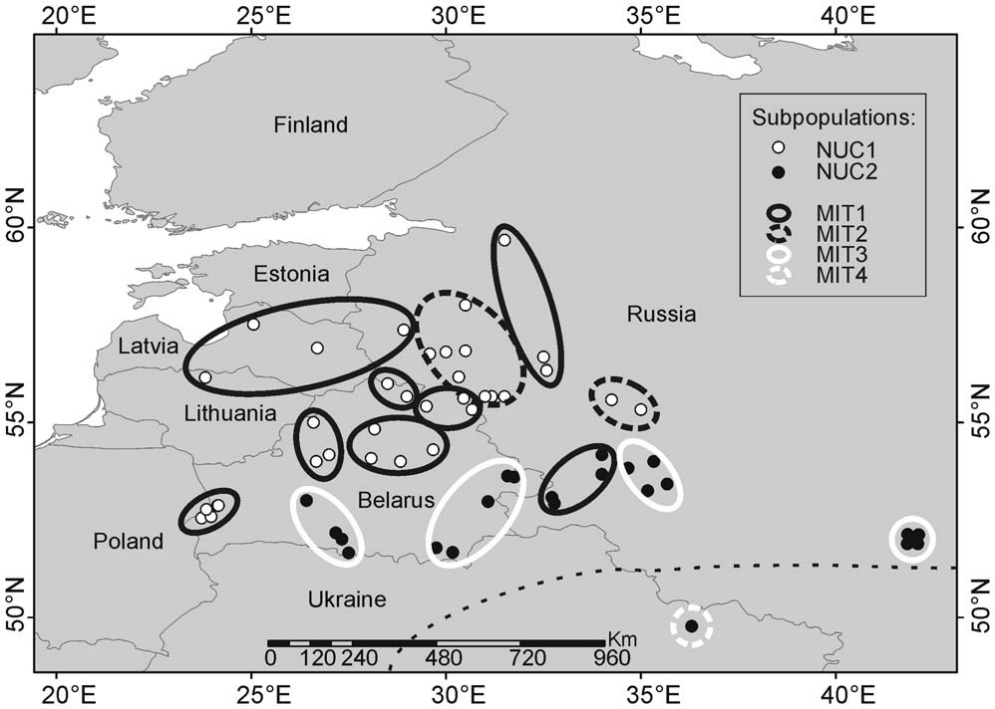
Geographic distribution of the grey wolf samples used in the stable isotope analysis. One point represents an individual or a group of individuals sampled in the same location, and large circles represent geographical regions the samples were grouped into. The samples were assigned to subpopulations delimited based on allele frequencies at 14 microsatellite loci (NUC 1 and NUC2), and frequencies of mtDNA haplotypes (MIT1-MIT4) based on the analysis of a larger dataset in Pilot *et al.* [7]. Dashed line represents the approximate border between two habitats: temperate mixed forest and forest-steppe.

Between 4 and 15 samples were analyzed from each geographical region, except for the southernmost region, CHAR, where only one sample was analyzed. Geographic distribution of the samples is presented in Figure 1. The number of samples analyzed per region was limited by the availability of the suitable material (i.e. muscle tissue samples preserved frozen). Other types of tissue could not be used in the comparative analysis due to differences in the isotopic turnover rate, and ethanol-preserved samples were not suitable for the analysis. As shown in the study on North American grey wolves, the sample sizes of n ≥4 should correctly reflect the stable isotope profiles of the local populations [17]. These results are consistent with another study performed for the black-tailed deer [18]. All samples were collected in the winter season (November-March) over a period of 9 years between 1995 and 2004. All individuals were adults or subadults, i.e. all were weaned. Individuals of both sexes were analyzed: 68 (62%) males and 42 (38%) females.

We analyzed muscle tissue samples which have a relatively fast turnover rate (a few months), because this allowed us to assess the diet obtained from an individual within a single locality, and to thereby avoid confounding signals from diet during earlier life stages (as would be reflected in bone collagen). Most wolves disperse from their natal packs, and they are able to move over distances up to 1000 km [19]. Therefore, using muscle tissue instead of bone collagen allowed us to control for geographic location when comparing isotopic variation against genetic diversity. The short time span represented in the muscle tissues is compensated by the fact that the samples from all but three regions were obtained in multiple years, and from a range of dates through the winter months. Therefore they represent averaged diet signals from several winter seasons and provide a good representation of the full winter season. Our data are thus season-specific yet time-averaged.

Additionally, in order to check whether stable isotope profiles accurately reflect wolf diet, we analyzed muscle tissue samples from the most common wolf prey species in Eastern Europe: moose *Alces alces* (5 individuals), red deer *Cervus elaphus* (5), roe deer *Capreolus capreolus* (5), wild boar *Sus scrofa* (4), beaver *Castor fiber* (5), and brown hare *Lepus europaeus* (9). These species accounted for a majority of biomass consumed by wolves in Eastern European ecosystems, as inferred from feces or stomach content composition [20]–[25]. The prey tissues were also sampled in Central-Eastern Europe, but did not cover all the geographic range of the wolf samples. Small sample size and geographic coverage of prey species (due to the limited availability of contemporary samples for this study) limits our resolution for determining diet composition, but this is not a primary objective for this study where our focus is instead on comparing a proxy to diet (wolf *δ* ^15^N and *δ* ^13^C stable isotope values) to population genetic structure.

### Sample Preparation and Stable Isotope Analysis

The samples were dried at 60°C for 48 h and homogenized with a mortar and pestle. To extract lipids, dried powdered samples were placed in glass tubes and immersed in 1.5 ml of 2∶ 1 mixture of chloroform : methanol. Samples were left in a shaker for 24 hours, then left undisturbed for 30 min, centrifuged for 10 min at 8 000 rpm and the supernatant was then discarded. This process was repeated at least three times or more until the supernatant was completely clear and colorless following centrifugation. Then samples were dried at 60°C for 24 h.

One mg of each sample was loaded into tin capsules. The samples were analyzed for their ^15^N and ^13^C isotopic composition using an automated nitrogen-carbon analyser (ANCA) coupled to a 20/20 isotope ratio mass spectrometer (IRMS) (SerCon Ltd, Crewe, UK). Two different analytical quality control samples (AQCs) were also analyzed with each batch for quality control purposes. These two AQCs were glutamic acid (*δ* ^15^N_Air_: −5.04‰ and *δ* ^13^C_VPDB_: −28.50‰) and leucine (*δ* ^15^N_Air_: 10.77‰ and *δ* ^13^C_VPDB_: −31.18‰). The international reference materials used for scale calibration were IAEA-CH6 (*δ* ^13^C_VPDB_ = −10.45‰, IAEA, Vienna, Austria), IAEA-600 (*δ* ^13^C_VPDB_ = −27.77‰, *δ* ^15^N_Air_ = 1.00‰, IAEA, Vienna, Austria) and USGS40 (*δ* ^13^C_VPDB_ = −26.39‰, *δ* ^15^N_Air_ = −4.52‰, IAEA, Vienna, Austria. International reference and standard materials for stable isotope analysis are administered, controlled, and issued by the Internal Atomic Energy Agency (IAEA, Vienna, Austria). Results of the isotopic analysis were expressed as *δ*-values relative to the international standards VPDB (Vienna-Pee Dee Belemnite) and Air (atmospheric N) for ^13^C and ^15^N, respectively. The analysis was replicated for 11 (10%) randomly selected samples. Each replicate was processed independently starting from the stage of sample powdering. The standard deviation for the replicates was <0.2‰ for *δ* ^13^C and <0.4‰ for *δ* ^15^N.

### Analysis of Wolf Diet Composition

The differences in isotopic abundance values for carbon and nitrogen among wolves in different geographical regions and subpopulations were evaluated using Kruskal–Wallis test. Relative consumption of different prey species by wolves was estimated using a dietary mixing model implemented in the program IsoSource [26]. IsoSource uses isotopic ratios to quantitatively determine the proportional contribution of several sources to a mixture, i.e. in this case the proportion of different prey species in the wolf diet. To calculate dietary endpoints corresponding to specific prey items, we used the mean isotopic signatures of muscles of each prey species corrected for dietary discrimination by adding the wolf-diet trophic enrichment values of 1.3±0.6‰ for *δ* ^13^C and 4.6±0.7‰ for *δ* ^15^N, estimated for Isle Royale wolves [17].

We also applied a Bayesian stable isotope mixing model implemented in the software MixSIR [27], which allowed us to account for uncertainty associated with multiple prey, and uncertainty in isotope values of each prey and the predator. Because we wanted to test how informative our data is, rather than precisely reconstruct the diet, we used uninformative priors. We performed 100,000,000 iterations in each run, which was sufficient to obtain a good performance of the model (see Table S3).

### Analysis of the Relationship between Diet Composition and Genetic Differentiation between Wolf Populations

Stable isotope abundance values were used as a quantitative measure of dietary differences between individuals and populations that could be related to genetic differentiation and geographical distances between them. This relationship was analyzed using a distance-based redundancy analysis, which is a form of multivariate multiple regression that can be performed directly on a genetic distance response matrix [28]. For the population-level analysis, we used a matrix of pair-wise *F*_ST_ values between geographical regions calculated based on the data on genetic variability of all 317 individuals from the earlier study [7]. The population-level DISTLM analysis was performed using the published microsatellite DNA data for local populations from 14 geographical regions (CHAR region with stable isotope data for one individual only was excluded from this analysis). For the individual-level analysis, we used pair-wise genetic distances between individuals calculated in GenAlEx [29]. For this analysis we used only the genetic data on the same individuals for which stable isotope values were measured. We used wolf diet composition represented by *δ* ^13^C and *δ* ^15^N values of tissue samples, and geographic distance (latitude and longitude) as predictor variables. We then tested two additional predictor variables: vegetation types and presence/absence of the moose. Moose was the only species for which geographical range did not cover the entire study area, so that the presence/absence criterion could be applied.

Using the program DISTLM v.5 [30], we performed the marginal tests on the correlation between stable isotope composition as a proxy for diet composition (hereafter referred to as ‘dietary differences’) and genetic distances between populations or individuals. We also performed the conditional test, where geographical distance was included as a co-variable. This allowed us to examine the extent to which dietary differences explain genetic diversification over and above that explained by geographic distance alone. We also performed the forward selection procedure on both sets of variables, using the program DISTLM *forward* [31]. The forward selection procedure consists of sequential tests, fitting each set of variables one at a time, conditional on the variables that are already included in the model. This approach allowed us to estimate the proportion of the genetic variation explained by both dietary differences and geographical distance, while controlling for their correlation.

Additionally, we analyzed the correlation between genetic distance and dietary differentiation between individuals and populations, using the Mantel test implemented in GenAlEx, which allowed us to graphically illustrate the dependence between these variables. In this analysis, we used the same measures of genetic distances between individuals and populations as in DISTLM analysis, a matrix of pair-wise genetic distances between individuals calculated in GenAlEx, and a matrix of isotopic distances between individuals, that was calculated by treating *δ* ^13^C and *δ* ^15^N values as two-dimensional Cartesian coordinates.

## Results

### Diet Composition of Eastern European Wolves

Mean *δ* ^15^N value for muscle tissue of Eastern European wolves was 9.3‰ (SD 1.0‰) and mean *δ* ^13^C value was −24.1‰ (SD 0.9‰). The ranges of stable isotope values were broad: from 5.97‰ to 12.14‰ for *δ* ^15^N and from −20.27‰ to −26.31‰ for *δ* ^13^C (for individual wolf results, see Table S1; for individual prey sample data, see Table S2). Wolves from different geographical regions significantly differed in stable isotope composition (*δ* ^15^N: H = 43.9, *P*<0.0001; *δ* ^13^C: H = 54.3, *P*<0.0001; Table 1). Wolves belonging to different subpopulations delimited based on mtDNA data (MIT1– MIT4) significantly differed in *δ* ^13^C values (H = 19.5, *P*<0.0001), while differences in *δ* ^15^N values were marginal (H = 3.7, *P* = 0.055; Table 2). Subpopulations delimited based on microsatellite data (NUC1 and NUC2) were significantly differentiated only for *δ* ^13^C values (H = 21.4, *P* = 0.0001; Table 2). Although mean isotopic differences among genetic subpopulations were modest, the underlying variation among 14 geographic regions (which was the basis on which we assessed the correlation between genetic and isotopic differentiation) was much larger, with *δ* ^15^N values ranging between 7.30‰ and 10.03‰ (difference 2.73‰), and *δ* ^13^C ranging between −25.79‰ and −23.19‰ (difference 2.60‰; see Table 1). There was no correlation between the year of collection and diet, as expressed by stable isotope values (R = 0.05, *P* = 0.60 for *δ* ^15^N and R = 0.18, *P* = 0.06 for *δ* ^13^C).

**Table 1:** Mean and standard deviation of *δ* ^15^N and *δ* ^13^C values (‰) for Eastern European wolves from 15 geographical regions.

Region	N	Location	Long	Lat	Mit pop	Nuc pop	mean *δ* ^15^N	mean *δ* ^13^C	SD *δ* ^15^N	SD *δ* ^13^C
MIN	5	N Belarus	28.48	54.30	1	1	9.87	−24.37	0.58	1.05
ROS	9	N Belarus/NW Russia	28.56	55.96	1	1	9.54	−24.49	0.41	0.65
VOL-POST	4	N Belarus	26.72	54.54	1	1	9.10	−24.54	1.64	0.55
VIT	10	N Belarus	30.07	55.41	1	1	10.03	−23.45	0.24	0.49
LAT	8	Latvia/NW Russia	26.99	57.05	1	1	9.15	−24.86	0.37	0.83
BIAL	5	NE Poland/E Belarus	23.97	52.73	1	1	7.30	−25.79	1.10	0.66
GAT	6	NW Russia	31.85	58.61	1	1	9.34	−23.96	1.77	0.64
MED-UNE	5	CW Russia	34.01	53.75	1	2	8.26	−24.05	0.12	0.52
SMO	9	NW Russia	33.69	55.81	2	1	9.70	−24.25	1.02	0.59
CHOLM	10	NW Russia	30.26	56.97	2	1	9.47	−24.52	1.05	0.75
GOM-MOG	15	S Belarus	30.29	52.05	3	2	9.21	−23.41	0.63	0.53
STO-GON	10	S Belarus	27.08	52.25	3	2	9.20	−23.62	0.52	0.79
KA-OREL	7	SW Russia	35.19	53.57	3	2	9.71	−23.19	0.39	0.57
TAMB	6	SW Russia	42.00	52.00	3	2	8.66	−24.30	0.34	0.21
CHAR	1	E Ukraine	36.30	49.77	4	2	11.35	−20.27	–	–

Mit pop: four subpopulations delimited based on mitochondrial DNA data.

Nuc pop: two subpopulations delimited based on 14 microsatellite loci.

BIAL region included individuals assigned to either subpopulations NUC1 or NUC2, and the region as a whole was assigned to subpopulation NUC1, where the majority of individuals were assigned.

**Table 2:** Mean values and standard deviation of *δ* ^15^N and d15N values (‰) for Eastern European wolves and their prey.

Group	N	mean *δ* ^15^N	mean *δ* ^13^C	SD *δ* ^15^N	SD *δ* ^13^C
Wolves					
MIT 1	52	9.07	−24.44	0.90	0.69
MIT 2	19	9.58	−24.38	0.16	0.19
MIT 3	38	9.19	−23.63	0.43	0.48
MIT 4	1	11.35	−20.27	–	–
NUC 1	66	9.28	−24.47	0.80	0.64
NUC 2	44	9.40	−23.14	1.08	1.46
Average	110	9.29	−24.05	0.97	0.95
Prey					
Moose	5	4.49	−26.97	1.35	0.76
Red deer	5	2.55	−27.01	1.58	1.20
Roe deer	5	3.69	−26.61	1.26	1.07
Wild boar	4	6.56	−20.95	1.07	3.12
Hare	9	5.81	−27.67	2.68	1.40
Beaver	5	6.25	−26.39	1.68	0.96

MIT 1-MIT 4: For the wolves, the average for all individuals is reported, as well as for four subpopulations delimited based on mtDNA data (MIT 1–4) and two subpopulations delimited based on microsatellite loci (NUC 1, NUC 2).

The composition of the wolf diet inferred in IsoSource based on the mean stable isotope values of the potential prey species is reported as 25th–75th percentile ranges for the relative contribution of each prey. The mean values are given for comparative purposes only and should be treated with caution [26]. The estimated average wolf diet in Eastern Europe consisted mainly of ungulates: red deer (range: 12–29%; mean: 20%), roe deer (9–34%; 22%), moose (5–23%; 15%), and wild boar (24–26%; 25%), with an admixture of beaver (3–12%; 8%) and hare (3–14%, 9%).

**Figure 2:**
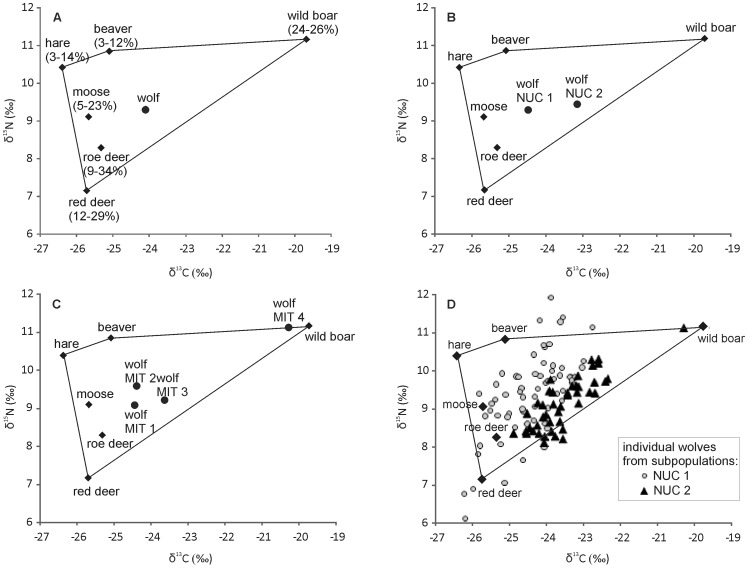
IsoSource dietary mixing polygon for Eastern European grey wolves. The wolf *δ* ^13^C and *δ* ^15^N values are plotted with potential prey. Trophic enrichment values of 1.3‰ for *δ* ^13^C and 4.6‰ for *δ* ^15^N [17] were added to the mean *δ* ^13^C and *δ* ^15^N values of potential prey. Stable isotope profiles are presented as mean and standard deviation for: (A) The entire wolf population. Contribution of each prey species to the diet is reported as the 25th to 75th percentile ranges of the estimated feasible distributions; (B) Subpopulations delimited based on microsatellite loci (NUC 1 and 2); (C) Subpopulations delimited based on mtDNA (MIT 1-4); (D) All analyzed individuals. Subpopulation MIT4 was represented by only one individual, and it was excluded from DISTLM analysis (see Materials and Methods). For standard deviation of prey stable isotope profiles, see Figure S2.

Significant differences in stable isotope values among wolf subpopulations in Eastern Europe were reflected in substantial differences in their inferred diet composition. The northern subpopulations (NUC1 incorporating MIT1 and MIT2) had a higher share of moose, hare and beaver in the diet as compared with the southern subpopulations (NUC2 incorporating MIT4 and most of MIT3), which had higher share of wild boar (Figure 2 and Table 3).

**Table 3:** Diet composition of wolves inferred from the stable isotope data using IsoSource for (A) subpopulations delimited based on mtDNA variability (MIT 1-MIT 4), (B) subpopulations delimited based on microsatellite variability (NUC 1, NUC 2), and (C) all individuals at average.

		Moose	Reddeer	Roedeer	Wildboar	Hare	Beaver
(A)							
MEAN	MIT 1	0.15	0.24	0.25	0.19	0.08	0.09
SD		0.12	0.12	0.18	0.02	0.06	0.07
25%ile		0.05	0.15	0.10	0.18	0.03	0.03
75%ile		0.23	0.34	0.38	0.20	0.12	0.13
MEAN	MIT 2	0.20	0.13	0.17	0.21	0.14	0.16
SD		0.16	0.09	0.12	0.02	0.09	0.11
25%ile		0.07	0.06	0.07	0.19	0.06	0.07
75%ile		0.29	0.20	0.26	0.22	0.20	0.23
MEAN	MIT 3	0.09	0.29	0.20	0.33	0.05	0.05
SD		0.08	0.10	0.15	0.02	0.04	0.04
25%ile		0.03	0.22	0.07	0.32	0.01	0.02
75%ile		0.14	0.37	0.30	0.34	0.07	0.08
MEAN	MIT 4	0.02	0.01	0.01	0.90	0.03	0.04
SD		0.02	0.01	0.01	0.01	0.02	0.03
25%ile		0.00	0.00	0.00	0.89	0.01	0.02
75%ile		0.02	0.01	0.02	0.90	0.05	0.06
(B)							
MEAN	NUC 1	0.18	0.19	0.22	0.19	0.10	0.12
SD		0.15	0.11	0.15	0.02	0.08	0.09
25%ile		0.07	0.10	0.09	0.17	0.04	0.05
75%ile		0.28	0.27	0.34	0.20	0.15	0.18
MEAN	NUC 2	0.08	0.27	0.17	0.41	0.04	0.04
SD		0.06	0.09	0.13	0.01	0.03	0.04
25%ile		0.02	0.21	0.06	0.40	0.01	0.01
75%ile		0.12	0.33	0.25	0.42	0.06	0.06
(C)							
MEAN	ALL	0.15	0.20	0.22	0.25	0.08	0.09
SD		0.12	0.11	0.16	0.02	0.06	0.07
25%ile		0.05	0.12	0.09	0.24	0.03	0.03
75%ile		0.23	0.29	0.34	0.26	0.12	0.14

We report mean, standard deviation (SD) and 25th–75th percentile (25 and 75%ile) ranges. The mean values are given for comparative purposes only and should be treated with caution because of the lack of uniqueness of the mixing model results [24]. The result for the subpopulation MIT 4 is based on one individual only and therefore is biased. This individual has not been considered in any population-based analyses.

The estimates of wolf diet from MixSIR had wider 25th–75th percentile ranges as compared with IsoSource, and in some cases multimodal distributions (see Table S3 and Figure S1), reflecting the uncertainty associated with dietary discrimination and isotope signatures, which was explicitly accounted for in this model [27]. This result is consistent with the results of the performance test of the MixSIR model, which showed that when sources had similar isotope signatures (as was the case with cervids in our study), the posterior distributions of source contributions exhibited strong multimodality [27]. Although the MixSIR results did not provide precise estimates of diet, they provided additional support for substantial differentiation between the northern and southern subpopulations, with higher share of wild boar and roe deer in the south, and higher share of hare in the north.

### Relationship between Dietary Differentiation, Geographical Distance and Genetic Differentiation among Individuals and Populations

The individual-based DISTLM analysis showed that genetic distances were highly correlated with both dietary differentiation and geographical distances (*P* = 0.0001 in each case). The conditional test showed that dietary differentiation was correlated with genetic distances over and above the influence of geographical distance (*P* = 0.0002). However, the forward selection procedure fitted geographical distance before dietary differentiation in the multiple regression model. Both variables altogether explained 10% of the genetic variability (Table 4). The moose presence/absence also was highly correlated with genetic distance (*P* = 0.0001), while no significant correlation was shown for the vegetation types. In the sequential test, both these variables were significant, and they were fitted after the stable isotopic measure of dietary differentiation in the multiple regression model, with the vegetation types being the least important variable (Table S4).

**Table 4:** Effects of dietary differentiation and geographic distance on genetic differentiation of Eastern European wolves.

	Marginal tests			Conditional tests			Sequential tests		
Variable set	pseudo-F	*P*	%var	pseudo-F	*P*	%var	pseudo-F	*P*	%var
(A) Individual-based test									
Coordinates	3.2	0.0001	5.9	–	–	–	3.2	0.0001	5.9
Stable isotope composition	2.5	0.0001	4.6	2.3	0.0003	4.1	2.3	0.0003	10.0
(B) Population-based test									
Stable isotope composition	4.7	0.005	46.1	4.0	0.033	28.7	4.7	0.005	46.1
Coordinates	3.6	0.019	39.3	–	–	–	3.1	0.034	68.0

Marginal and conditional tests of individual variable sets as well as sequential tests of the forward selection procedure are reported (see Methods for the description of the tests). “Pseudo-F” indicates test statistics, *P* probability values and “%var” the percentage of the genetic variation explained by the particular variable. In the case of sequential tests, “%var” indicates the percentage of the genetic variation explained by a cumulative effect of variables. The top-down sequence of variables corresponds to the sequence that was indicated by the forward selection procedure. The variable set “coordinates” included latitude and longitude, and “stable isotope composition” included *δ* ^15^N and *δ* ^13^C values. Genetic distances were calculated based on the data on variability at 14 microsatellite loci obtained in an earlier study [7].

The population-level DISTLM analysis showed that genetic distances between local populations were correlated with both dietary differentiation (*P* = 0.005) and geographical distances (*P* = 0.019), and they explained 46% and 39% of the variation, respectively. The conditional test showed that dietary differentiation was correlated with genetic distances over and above the influence of geographical distance (*P* = 0.033) and explained 29% of variation. The forward selection procedure fitted dietary differentiation before geographical distance in the multiple regression model. Both variables altogether explained 68% of the genetic variability (Table 4). When latitude and longitude were considered as two separate variables, only latitude was correlated with the genetic distance (P = 0.03), suggesting that the correlation is due to environmental factors that in Europe change along the latitudinal gradient rather than geographical distance alone (see [7]). Accordingly, stable isotope differentiation was fitted before latitude by the forward selection procedure (P = 0.02). We also assessed the correlation between isotopic variation among local populations and geographic distance while controlling for genetic distance. This conditional test showed no significant correlation (*P* = 0.34), suggesting that there is little variation in the stable isotope signal due to geographic distance alone.

Moose presence/absence also was highly correlated with the genetic distance (*P* = 0.0006) and explained 31.5% of the genetic variation. However, in the sequential test, this variable was fitted after the dietary differentiation and geographical distance, and was non-significant (Table S4). Vegetation types were not significantly correlated with genetic distances, and did not increase the genetic variation explained by the predictor variables in the sequential test. When considered separately, *δ* ^13^C and *δ* ^15^N explained a similar proportion of genetic variability (28% for *δ* ^13^C, *P* = 0.02 and 25% for *δ* ^15^N, *P* = 0.04), and differentiation between geographic regions calculated separately for *δ* ^13^C and *δ* ^15^N was significantly correlated (R = 0.61, *P* = 0.02).

**Figure 3:**
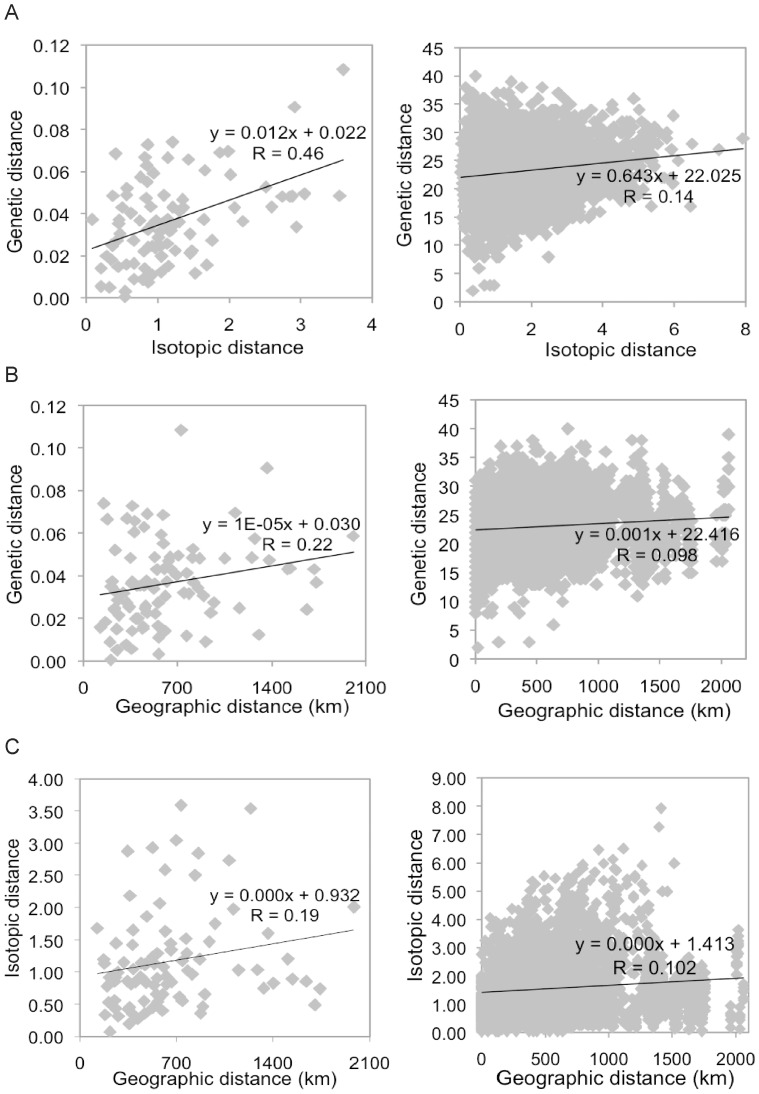
Graphical illustration of correlations between genetic and dietary differentiation and geographic distance. Correlations are presented at a population (left) and individual (right) level. (A) The correlation between genetic and stable isotope differentiation. Genetic distances between populations were represented by pairwise *F*_ST_ values. Genetic distances between individuals were calculated using a method implemented in GenAlEx. Isotopic distances between populations and individuals were calculated by treating *δ* ^13^C and *δ* ^15^N values as two-dimensional Cartesian coordinates. Both correlations are significant (see Results). (B) The correlation between genetic and geographic distances. Only the correlation at individual level is significant (*P* = 0.04). (C) Correlation between stable isotope differentiation and geographic distances. Subpopulation MIT4 was represented by only one individual, and it was excluded from the population-level analysis.

The Mantel test confirmed the significant correlation of genetic distances with dietary differentiation at both individual (*P* = 0.008) and population level (*P* = 0.045), and also showed that at the population level dietary differentiation explains a large percent of genetic variation (R = 0.46; Figure 3). There was a marginal correlation between isotopic and geographic distances, not significant at the 0.05 level (Mantel test, R=0.102, P=0.06 for individual-based data and R=0.205, P=0.10 for populations Figure 3).

## Discussion

Both individual-based and population-based analyses consistently showed a correlation between dietary and genetic differentiation. While individual dietary differences explained a comparatively small percentage of the genetic variation (which could be due to strong individual variation in wolf diet, e.g. [32]), the population-level data showed that dietary differences explain a large proportion of genetic differentiation. This association remained significant (with the largest proportion of the genetic variance explained by the diet) after the influence of geographical distance was accounted for. This suggests that ecological factors determining wolf diet (e.g. habitat type and the associated composition of the ungulate community) are contributing to the structuring of wolf populations. We discuss this in the context of alternative interpretations after considering the utility of our stable isotope data as a reliable marker of dietary differentiation in wolves across the study range.

### Reliability of Stable Isotope Data as a Proxy for Wolf Diet

In our study, the mean *δ* ^13^C value for muscle tissue in Eastern European wolves was the same order as that reported for North American wolves from Minnesota [17]. However, the mean *δ* ^15^N value in North American wolves was 2.1‰ lower. This may result from the lack of wild boar (which as an omnivore has higher tissue *δ* ^15^N values than primary consumers such as cervids and hares) from the diet of North American wolves, and from lower *δ* ^15^N values of the same or sister prey species in North America as compared to Europe [17], [32]. The broad ranges of stable isotope values of Eastern European wolves suggested a highly varied diet and dietary differences among individuals, which is consistent with the results of stable isotope studies on North American wolves [17], [32].

Some portion of wolf isotopic variability potentially could be due to geographic variation (e.g. resulting from variation in plant or soil isotopic values) rather than dietary niche differences between populations. For example, there is an observed gradient in *δ* ^13^C values for C_3_ and C_4_ terrestrial plants over broad latitudinal ranges [33], most pronounced in C_4_ plants. However, the trophic relationships relevant to our study will be dominated by C_3_ plants at the base, and do not show a trend over the relevant latitudinal range (50°–60° North) in C_3_ plants [33], herbivores or carnivores [34]. There is also the potential for a ‘canopy effect’ leading herbivores in open habitat to have different isotopic values than those in forested habitat [35]. However, red and roe deer compared among relevant geographic regions in Europe showed no clear difference [35], [36], and no evidence for a simple canopy effect was found in European red deer [36]. Further, a key aspect of the results was the lack of significant correlation between stable isotope signal and geographic distance (see Figure 3), which would not be expected if the signal for a given prey species varied with geographic distance (or would require some unlikely counterbalancing of signals among prey species). We also showed that there was no correlation between isotopic values and geographic distance when we controlled for genetic diversity.

Lack of evidence for the correlation between stable isotope composition of wolf tissues and geographic distance is consistent with studies suggesting that variation in ungulate stable isotope signal is not an important factor over the relevant geographic range. We cannot exclude the possibility that plant or soil isotopic values may vary in a non-linear manner, independent of geographical distance, but such variation is much more likely to decrease the strength of the correlation between genetic differentiation and the inferred dietary differentiation than to produce a false signal for a significant correlation. Furthermore, for non-linear variation due to factors other than diet, we could expect inconsistent patterns between the two isotopes (see [34]). However, in our study differentiation calculated separately for *δ* ^13^C and *δ* ^15^N is significantly correlated, and each of these two variables separately explains a similar proportion of the genetic variability.

Stable isotope composition of muscle tissue reflects the diet within a period of several weeks [37], and so seasonal changes in wolf or prey diet [38], [39] could potentially affect the results. We controlled for this by sampling individuals in the winter season only, and there is no evidence for significant variability of wolf diet during the winter season in Eastern Europe [20], [22], [24], [25], [40]. Moreover, all but three regions were sampled in multiple years, so the results represent average diet over several winter seasons. However, we found no correlation between the year of sample collection and diet, as expressed in stable isotope values.

If seasonal patterns or variation in the stable isotope signal of prey species among the geographic regions covered by this study were significantly confounding our analyses, the effect should be to disrupt our ability to detect a particular prey species in multiple regions, and the apparent prey composition in the estimated diets should be inconsistent with data from other sources. The average wolf diet in Eastern Europe during the winter season as estimated from our stable isotope data consisted mainly of ungulates, with an admixture of beaver and hare. Stable isotope studies on North American wolves indicated similar prey choice [17], [32]. This result is also consistent with the studies on wolf feeding ecology in Eastern Europe based on traditional methods such as observational data, stomach and scat analyses [20]–[25], [40].

The differences in the diet composition between local populations estimated using IsoSource also are consistent with these earlier studies, as well as with the data on prey species distribution (though we have not tested for and do not mean to imply a direct match with relative abundance): In north-eastern Europe, the moose is an important part of the ungulate community (in terms of frequency and biomass), and it is an important wolf prey [20], [41]. In the middle latitudes of Eastern Europe, where the moose is less common, and the red deer, roe deer, and wild boar dominate in the ungulate community, a positive selectivity for the red deer and strong functional response to an increase in red deer densities have been observed [21], [22]. In southern Europe, where the moose does not occur and the red deer is less abundant, the roe deer and the wild boar dominate in the wolf diet, and in some locations the wild boar is the only wild prey of wolves [42], [43]. Overall the data reflect a pattern of prey choice that is credible based on earlier studies. Various factors may be important in the choice of a particular set of prey in a given location, including but not restricted to relative abundance. As is evident from our IsoSource analyses, the different genetic populations (e.g. MIT1-3) showed diets that included different proportions of key prey species (see Table 3). These patterns were consistent with but not strictly determined by the geographic distributions of the prey. For example, populations MIT1 and MIT2 are distributed across a similar geographic range (Figure 1) and yet their apparent proportional take of different prey species varies (Table 3).

In contrast to IsoSource, the MixSIR model did not give precise estimates of wolf diet due to multimodal probability distributions of prey composition. This was the effect of explicitly incorporating the uncertainty associated with dietary discrimination and isotope signatures, and analysing prey with similar isotope profiles [27]. A simulation study showed that results from the MixSIR model converge to IsoSource results when sources of uncertainty are reduced [27]. In our study, the main source of uncertainty was a large variance of prey isotopic signatures resulting from small sample sizes, and therefore more comprehensive prey sampling is needed to obtain precise diet estimates from this model. However, even our limited prey dataset provided a clear support for substantial differentiation between the wolf subpopulations from the northern and southern part of our study area.

### Patterns of Genetic Differentiation

Geographic distance is a common factor associated with genetic differentiation. In this study however, correlation data in support of isolation by distance was weak. For example, large geographic distances from east to west sometimes showed low differentiation while small distances north to south showed stronger differentiation. Even when considering latitude and longitude separately, latitude (which unlike longitude showed a significant pattern) still explained less of the genetic variance than the stable isotope data. Our correlations with geographic distance considered straight-line distances, but there are no credible impediments to wolf dispersal over the study range that would suggest a more appropriate pathway [7]. Within the study area, there are no altitudinal barriers (elevation across the range varies from zero to about 200 m), most river systems in the region run north to south (and freeze during the winter), and forest habitats are interconnected and form a continuous network. A further possibility is historical vicariance and isolation in allopatry, followed by reconvergence. However, phylogeographic analyses based on mtDNA data do not support such a scenario [7], [44]. Our data show a consistent relationship between genetic differentiation and isotopic values (a proxy for diet) that was greater than correlation with geographic distance, which suggests that ecological factors are important in defining these genetic populations.

The level of neutral genetic differentiation among populations reflects the long-term patterns of effective dispersal, and an association between non-random dispersal based on prey or habitat preferences and genetic differentiation is a likely mechanism. The preference of dispersing individuals toward habitats similar to their natal habitat has been observed in a broad range of animal species [16]. Various authors have proposed that these types of preferences may translate into discretely subdivided populations along physically unobstructed habitat boundaries [3], [6], [7], [12]. Such cryptic population structure was observed in various carnivorous mammals [1], [4], [6], [12], including grey wolves from North America [3], [8]–[10] and Eastern Europe [7]. In carnivores, the availability (i.e. presence, abundance, and conditions affecting hunting success) of familiar prey species is considered as an important habitat cue (e.g. [2], [7], [8]). In the grey wolf, it was demonstrated that learning and experience improve hunting success [45], which may result in individual preferences for particular prey types (though wolves are known to be able to adjust to new prey in some cases, e.g. after translocations). These preferences may affect dispersal decisions, either directly or indirectly (by choosing habitats with familiar characteristics that are correlated with the prey composition). As reviewed in the introduction, this relationship between prey choice and dispersal is also found in various other species, such as other carnivores and cetaceans. It has even been proposed as a mechanism for directed dispersal in micro-organisms (see review in [46]).

The key role of diet in shaping evolutionary processes in carnivores was also indirectly inferred based on genetic and isotopic analyses of late Pleistocene and early Holocene grey wolves. It has been shown that the Pleistocene wolves in North America and Europe mainly preyed on megafaunal species like large bovids and horses 47,48, and the substantial decline of these prey species at the beginning of the Holocene coincided with complete (North America) or partial (Europe) replacement of wolf mtDNA lineages 44,47.

In our study we use a metric related to diet that can be quantified from the same individuals that were also genotyped. This allows a direct comparison between the genetic differentiation among individuals and their diet (to the extent that diet is well represented by the stable isotopic values). We find a stronger signal for diet correlated to genetic distance than found in an earlier study on the same populations [7], and propose that this is due to the greater precision made possible from the stable isotope data (which will reflect total individual diet rather than regional average prey composition) and from the inclusion of data for both metrics from the same set of individual animals. There is some signal for a correlation with geographic distance, as found in an earlier study [7], and other factors are also likely to be affecting our results (such as noise from an imprecise relationship between stable isotope signal and diet, as discussed above).

We also found a significant correlation between genetic distance and presence/absence of the moose, which supports the hypothesis that differences in availability of particular prey types constitute an important factor shaping genetic differentiation in wolf populations. However, the forward selection procedure in DISTLM classified this variable as less important than stable isotope data, suggesting that overall dietary differentiation explains wolf genetic variability better than the presence/absence of any single species. Vegetation types were not correlated with genetic variability, unlike in the earlier study [7]. However, the earlier study included samples from a wider geographical area, spanning several different habitats, while the present study area included only two habitat types: temperate mixed forest and forest-steppe. This result suggests that within similar habitats, genetic differentiation of wolves may depend directly on differences in prey composition, likely associated with microhabitat differentiation affecting herbivore species distribution and abundance.

The integration of ecology and evolution is a necessary step towards major advances in our understanding of the processes that shape and maintain biodiversity [49]. Our data provide evidence based on carefully controlled correlations that support a growing literature indicating a relationship between prey or habitat choice and population genetic structure. The inference is consistent and clear, however further data could help determine that there is a causative relationship. The next step towards understanding this system should include tracking studies where individual foraging behavior in natal and post-dispersal locations can be assessed. It may be also worth testing whether the observed patterns are associated with local differential selection.

## Supporting Information

Figure S1 **Distributions of posterior estimates of proportional contributions of prey sources in diet of Eastern European wolves inferred from the stable isotope data using MixSIR for: four subpopulations delimited based on mtDNA variability (MIT 1-MIT 4), two subpopulations delimited based on microsatellite variability (NUC 1, NUC 2), and the entire population.**
(PDF)

Figure S2 **IsoSource dietary mixing polygon for Eastern European grey wolves.** The wolf *δ*13C and *δ*15N values are plotted with potential prey. Trophic enrichment values of 1.3‰ for *δ*13C and 4.6‰ for *δ*15N (from Fox-Dobbs *et al.* 2007) were added to the mean *δ*13C and *δ*15N values of potential prey. Stable isotope profiles are presented as mean and standard deviation for the wolf and each prey species.
(PDF)

Table S1 **Sample information and *δ*15N and *δ*13C isotope profiles (‰) for 110 Eastern European wolves analyzed in this study.**
(PDF)

Table S2 **Sample information and *δ*15N and *δ*13C isotope profiles (‰) for wolf prey species analyzed in this study.**
(PDF)

Table S3 **Diet composition of wolves inferred from the stable isotope data using MixSIR.** For: (A) four subpopulations delimited based on mtDNA variability (MIT 1- MIT 4), (B) two subpopulations delimited based on microsatellite variability (NUC 1, NUC 2), and (C) all individuals pulled together. The result for the subpopulation MIT 4 is based on one individual only and therefore is biased. We report median and 25th–75th percentile (25 and 75%ile) ranges. Because in some cases the posterior distributions are multimodal, these percentiles may not adequately describe the posterior surface of the source contributions. The posterior distributions are presented in the Figure S1.
(PDF)

Table S4 **Effects of ecological factors and geographical distance on genetic differentiation of Eastern European wolves.** The tests were performed in the same way as those presented in Table for, but additional explanatory variables were considered. For details see Table 4 legend and Methods section with the description of the tests.
(PDF)
