# Supplementary material for: Dietary Differentiation and the Evolution of Population Genetic Structure in a Highly Mobile Carnivore
Source: PLoS One. 2012 Jun 29;7(6):e39341. doi: 10.1371/journal.pone.0039341 (PMC3387138; doi:10.1371/journal.pone.0039341)

Figure S1. Distributions of posterior estimates of proportional contributions of prey sources in diet of Eastern European wolves inferred from the stable isotope data using MixSIR for: four subpopulations delimited based on mtDNA variability (MIT 1-MIT 4), two subpopulations delimited based on microsatellite variability (NUC 1, NUC 2), and the entire population.

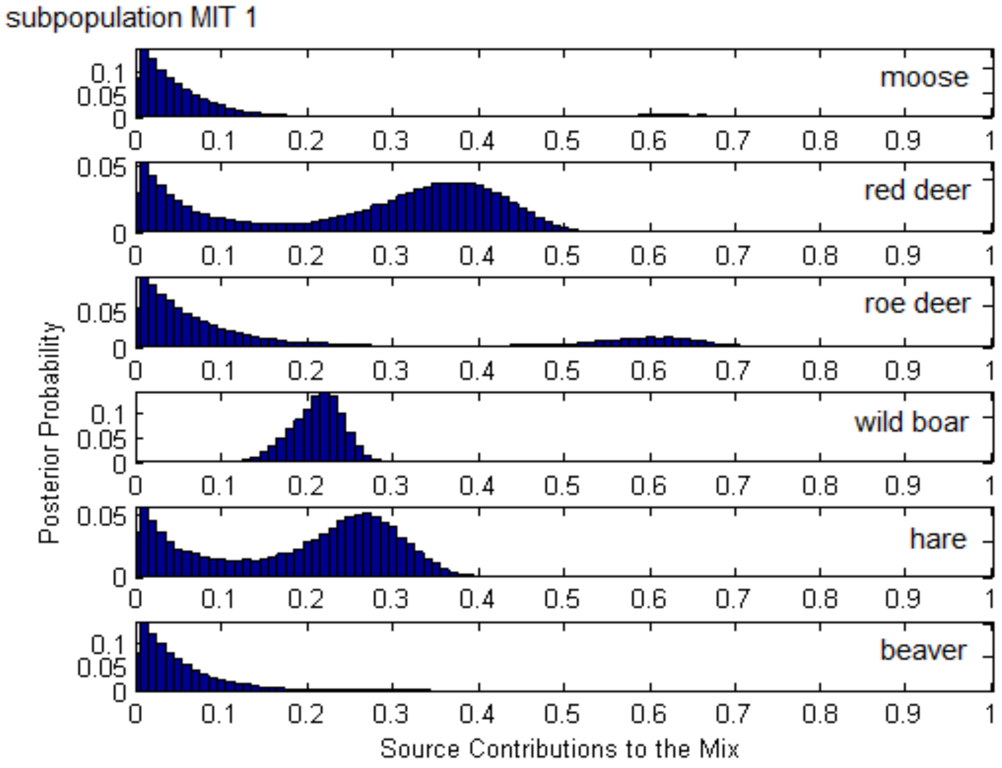

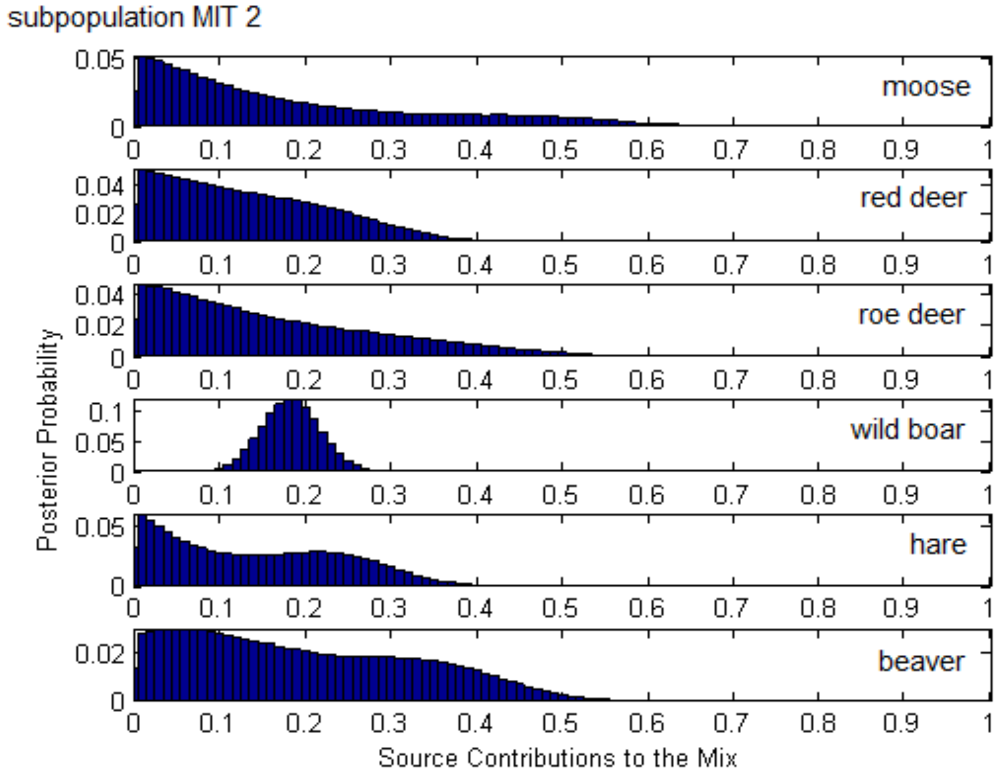

subpopulation MIT 3

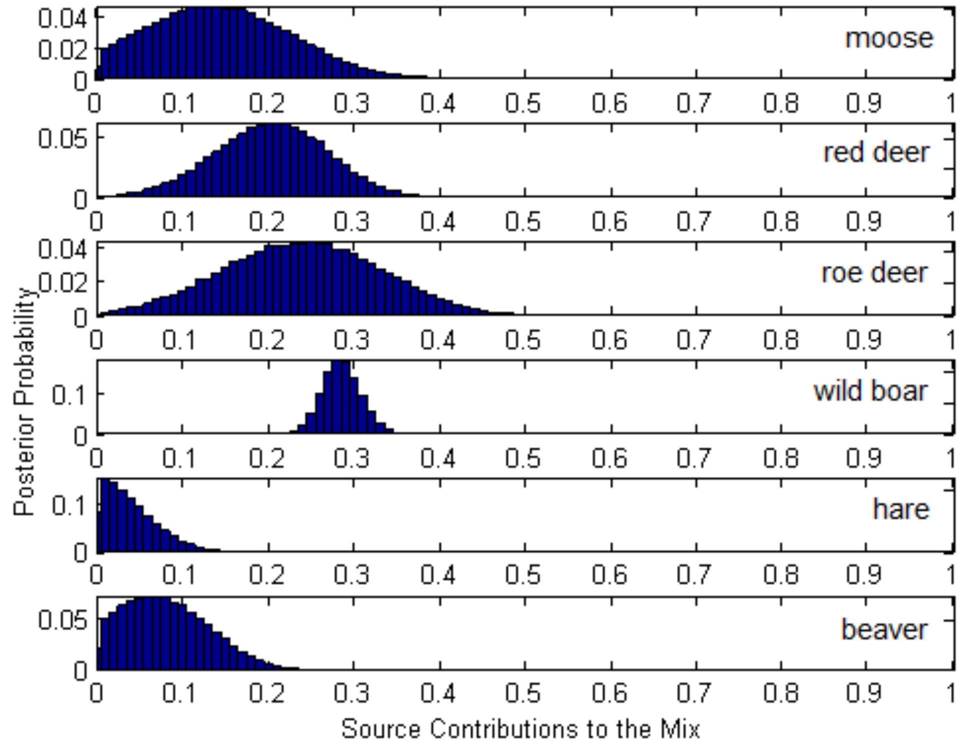

subpopulation MIT 4

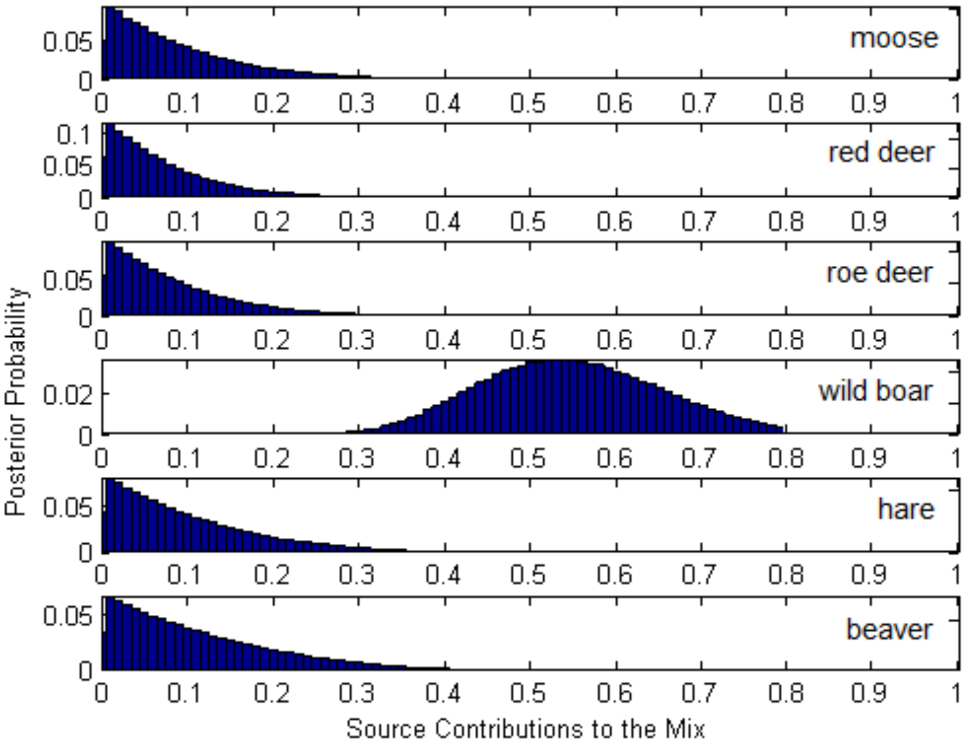

subpopulation NUC 1

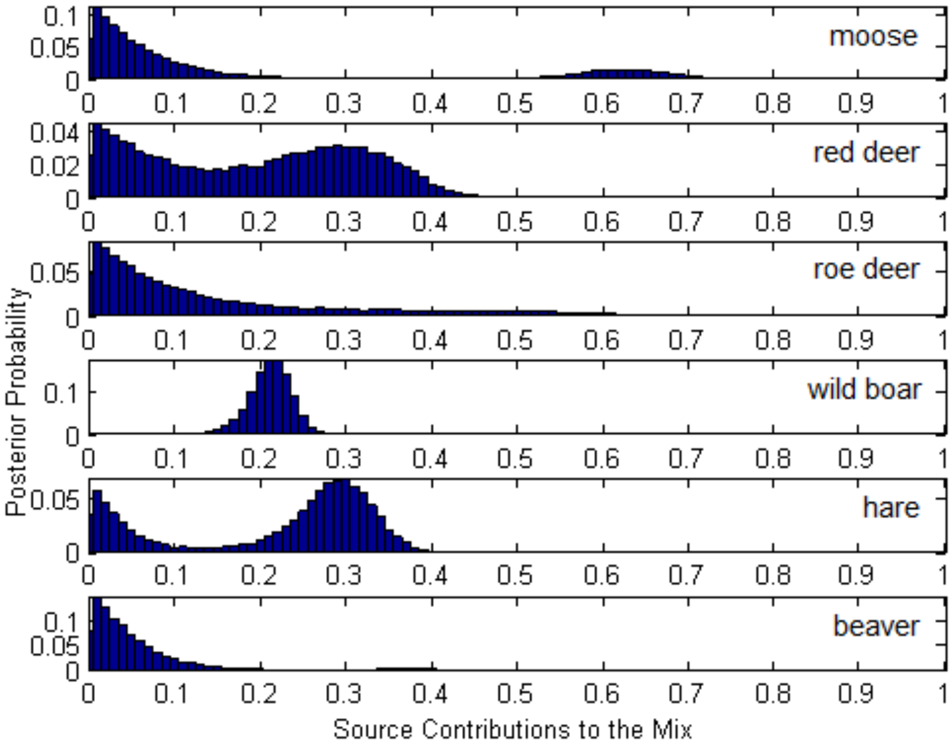

subpopulation NUC 2

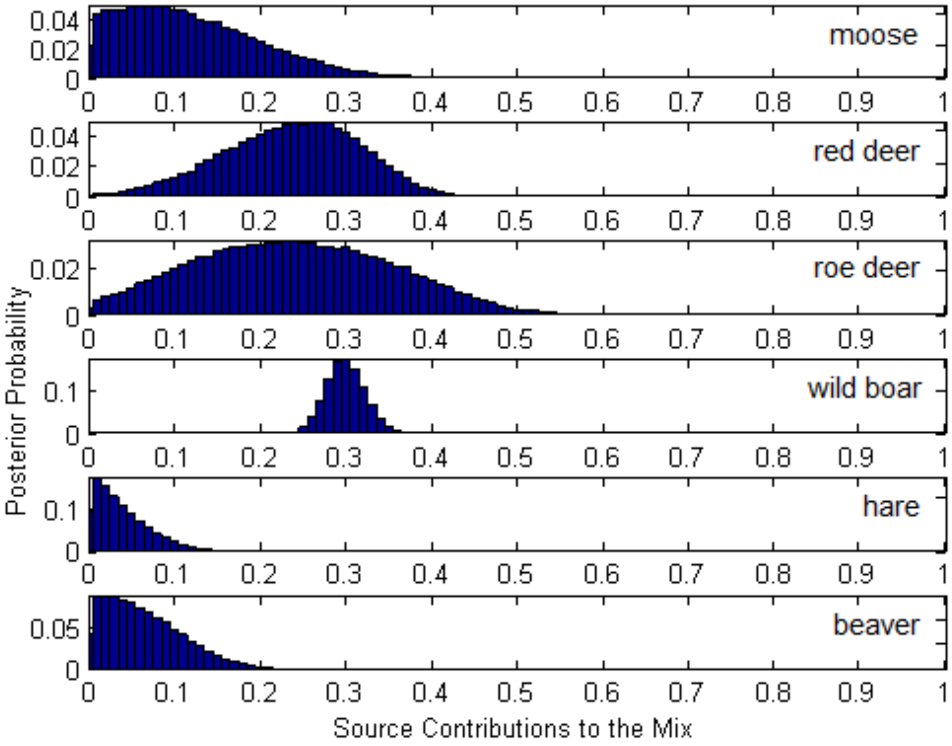

entire population

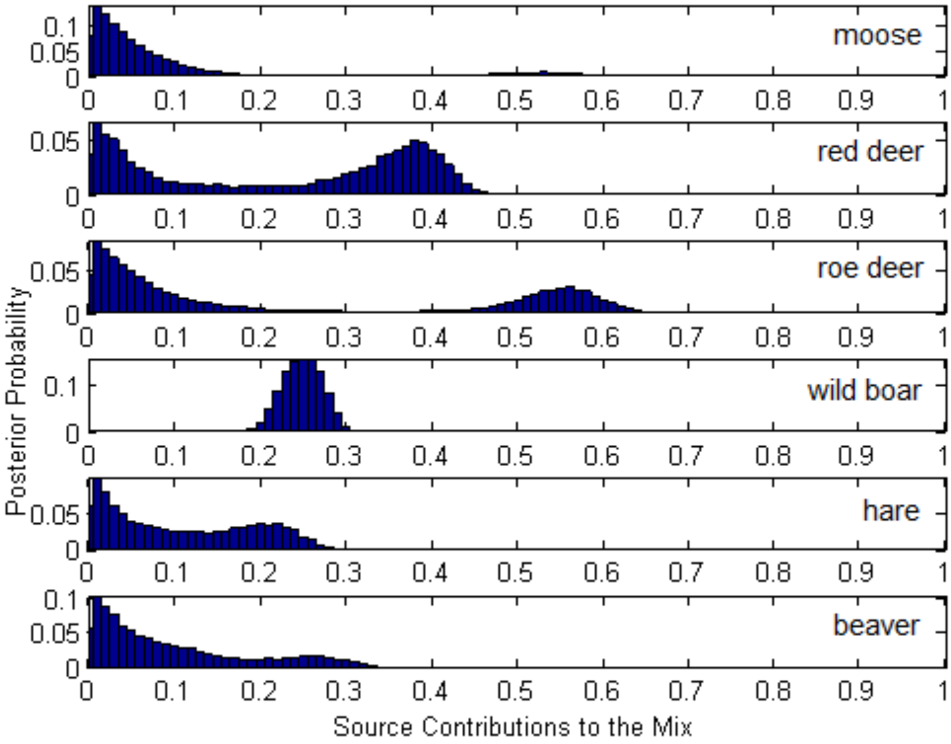

Supplement: Figure S1 — Distributions of posterior estimates of proportional contributions of prey sources in diet of Eastern European wolves inferred from the stable isotope data using MixSIR for: four subpopulations delimited based on mtDNA variability (MIT 1-MIT 4), two subpopulations delimited based on microsatellite variability (NUC 1, NUC 2), and the entire population. (PDF) [file pone.0039341.s001.pdf]
