# Supplementary material for: Dietary Differentiation and the Evolution of Population Genetic Structure in a Highly Mobile Carnivore
Source: PLoS One. 2012 Jun 29;7(6):e39341. doi: 10.1371/journal.pone.0039341 (PMC3387138; doi:10.1371/journal.pone.0039341)

Figure S2. IsoSource dietary mixing polygon for Eastern European grey wolves. The wolf $\delta^{13}C$ and $\delta^{15}N$ values are plotted with potential prey. Trophic enrichment values of 1.3‰ for $\delta^{13}C$ and 4.6‰ for $\delta^{15}N$ (from Fox-Dobbs *et al.* 2007) were added to the mean $\delta^{13}C$ and $\delta^{15}N$ values of potential prey. Stable isotope profiles are presented as mean and standard deviation for the wolf and each prey species.

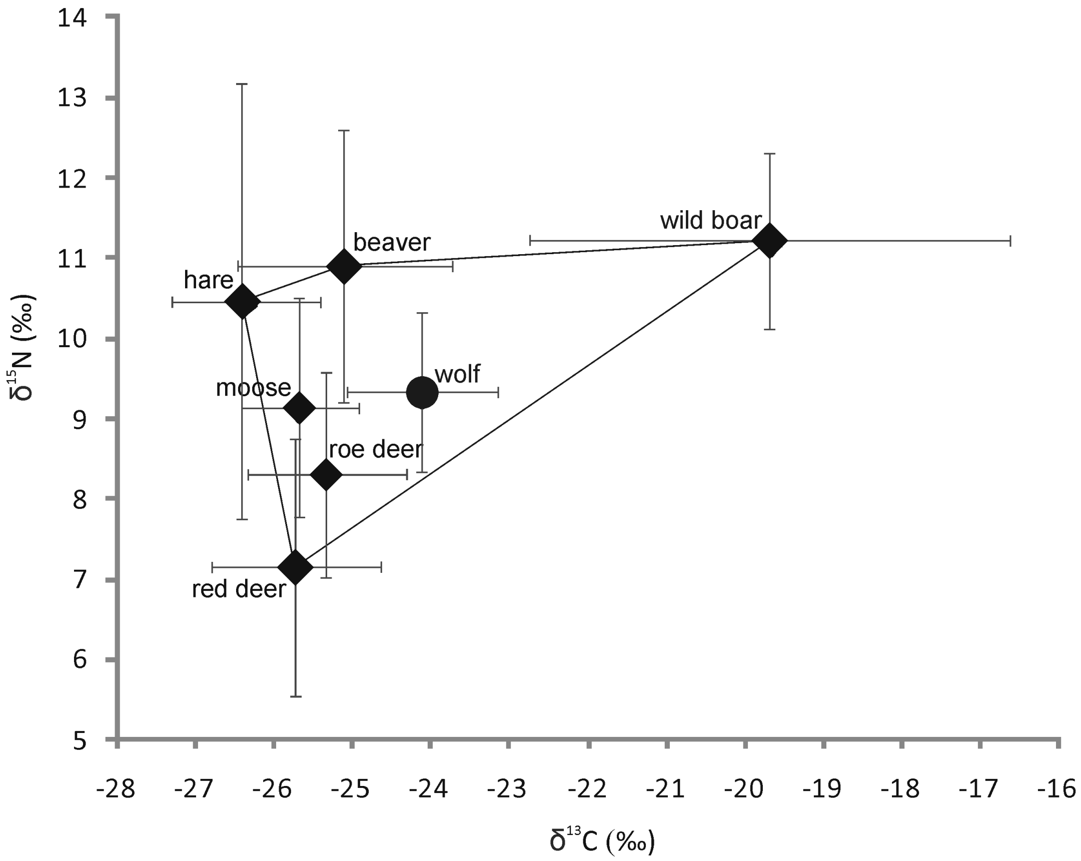

Supplement: Figure S2 — IsoSource dietary mixing polygon for Eastern European grey wolves. The wolf δ13C and δ15N values are plotted with potential prey. Trophic enrichment values of 1.3‰ for δ13C and 4.6‰ for δ15N (from Fox-Dobbs et al. 2007) were added to the mean δ13C and δ15N values of potential prey. Stable isotope profiles are presented as mean and standard deviation for the wolf and each prey species. (PDF) [file pone.0039341.s002.pdf]
