# Supplementary material for: Dietary Differentiation and the Evolution of Population Genetic Structure in a Highly Mobile Carnivore
Source: PLoS One. 2012 Jun 29;7(6):e39341. doi: 10.1371/journal.pone.0039341 (PMC3387138; doi:10.1371/journal.pone.0039341)
Supplement: Table S1 — Sample information and δ15N and δ13C isotope profiles (‰) for 110 Eastern European wolves analyzed in this study. (PDF) [file pone.0039341.s003.pdf]

Table S1. Sample information and $\delta^{15}N$ and $\delta^{13}C$ isotope profiles (‰) for 110 Eastern European wolves analysed in this study.

Mit subpop - subpopulation assignment based on mtDNA data; Nuc subpop - subpopulation assignment based on microsatellite loci; Region – spatial grouping of individuals used for population-level analysis; Age class: ad – adult, sad – subadult, U – unknown (either adult or subadult).

Sample ID	$\delta^{15}N$	$\delta^{13}C$	Longitude	Latitude	Region	Mit subpop	Nuc subpop	Season	Sex	Age class
14	6.76	-26.23	24.14	52.86	BIAL	1	1	95/96	F	sad
16	7.80	-25.86	24.16	52.87	BIAL	1	1	96/97	F	sad
31	8.94	-24.63	23.73	52.55	BIAL	1	1	98/99	F	U
45	6.88	-25.99	23.97	52.58	BIAL	1	1	99/00	M	sad
50	8.40	-24.38	31.58	53.63	GOM-MOG	3	2	99/00	M	U
51	9.44	-22.85	29.75	51.78	GOM-MOG	3	2	99/00	F	U
52	8.45	-23.58	29.75	51.78	GOM-MOG	3	2	99/00	M	U
53	8.39	-23.89	32.72	53.08	MED-UNE	1	2	99/00	M	U
54	8.34	-24.22	31.08	52.97	GOM-MOG	3	2	99/00	F	U
55	8.66	-23.66	30.17	51.67	GOM-MOG	3	2	99/00	M	U
56	8.64	-23.93	31.75	53.60	GOM-MOG	3	2	99/00	M	U
64	9.00	-24.66	25.00	57.60	LAT	1	1	98/99	F	U
66	8.81	-25.68	23.80	56.08	LAT	1	1	98/99	F	U
71	9.24	-25.41	26.70	56.90	LAT	1	1	99/00	F	U
72	9.37	-25.12	26.70	56.90	LAT	1	1	99/00	M	U
73	9.40	-25.84	26.70	56.90	LAT	1	1	99/00	F	U
83	9.94	-25.22	28.50	56.00	ROS	1	1	99/00	F	U
84	9.22	-23.60	28.50	56.00	ROS	1	1	99/00	F	U
85	8.95	-25.55	28.50	56.00	ROS	1	1	99/00	M	U
93	9.80	-23.80	29.00	55.67	ROS	1	1	99/00	M	U
99	10.15	-22.76	30.17	51.67	GOM-MOG	3	2	99/00	M	U
100	10.72	-23.93	29.67	54.30	MIN	1	1	99/00	F	U
101	9.65	-25.38	29.67	54.30	MIN	1	1	99/00	F	U
103	9.60	-23.21	29.75	51.78	GOM-MOG	3	2	99/00	F	U
105	9.87	-23.17	30.17	51.67	GOM-MOG	3	2	99/00	M	sad
113	9.41	-22.70	29.75	51.78	GOM-MOG	3	2	99/00	M	U
115	9.06	-23.64	29.75	51.78	GOM-MOG	3	2	99/00	F	U
116	10.00	-23.87	30.67	55.33	VIT	1	1	99/00	M	U
117	10.08	-23.59	30.67	55.33	VIT	1	1	99/00	M	U
118	10.27	-23.00	30.67	55.33	VIT	1	1	99/00	F	U
119	8.91	-23.70	30.17	51.67	GOM-MOG	3	2	99/00	M	U
120	9.83	-23.36	30.67	55.33	VIT	1	1	99/00	F	U
122	9.36	-23.45	30.17	51.67	GOM-MOG	3	2	99/00	M	U
125	10.30	-22.79	30.17	51.67	GOM-MOG	3	2	99/00	M	U
126	6.11	-26.22	23.85	52.77	BIAL	1	1	00/01	F	ad
133	9.85	-23.38	29.50	55.42	VIT	1	1	00/01	M	U

135	9.68	-23.31	29.50	55.42	VIT	1	1	00/01	M	U
136	10.18	-22.59	29.50	55.42	VIT	1	1	00/01	M	U
138	9.83	-23.25	29.50	55.42	VIT	1	1	00/01	M	U
139	10.14	-23.85	29.50	55.42	VIT	1	1	00/01	M	U
142	9.86	-24.18	31.17	55.67	SMO	2	1	00/01	F	U
143	9.53	-24.24	31.17	55.67	SMO	2	1	00/01	M	U
144	9.70	-24.03	31.17	55.67	SMO	2	1	00/01	M	U
163	8.07	-25.25	30.00	56.80	CHOLM	2	1	00/01	M	U
165	8.09	-24.08	32.78	52.92	MED-UNE	1	2	00/01	M	U
166	10.10	-23.05	28.17	54.83	MIN	1	1	00/01	M	U
168	9.15	-25.50	28.08	54.08	MIN	1	1	00/01	F	U
169	8.34	-24.91	32.78	52.92	MED-UNE	1	2	00/01	F	U
184	8.82	-25.33	31.00	55.67	SMO	2	1	00/01	M	U
185	9.12	-23.84	27.33	52.00	STO-GON	3	2	00/01	M	U
190	11.33	-24.20	31.00	55.67	SMO	2	1	00/01	M	U
193	8.88	-25.03	30.00	56.80	CHOLM	2	1	00/01	M	U
196	9.86	-24.82	28.50	56.00	ROS	1	1	00/01	M	U
197	9.80	-22.35	27.33	52.00	STO-GON	3	2	00/01	M	U
198	9.92	-24.31	28.50	56.00	ROS	1	1	00/01	F	U
199	9.84	-24.77	28.50	56.00	ROS	1	1	00/01	M	U
200	9.02	-23.97	28.50	56.00	ROS	1	1	00/01	M	U
201	9.14	-23.15	27.33	52.00	STO-GON	3	2	00/01	M	U
203	9.58	-23.15	30.17	51.67	GOM-MOG	3	2	00/01	M	U
205	8.84	-24.03	27.33	52.00	STO-GON	3	2	00/01	M	U
206	9.32	-24.33	28.50	56.00	ROS	1	1	00/01	F	U
225	9.09	-24.12	27.17	52.17	STO-GON	3	2	00/01	F	sad
235	9.70	-22.72	35.33	54.00	KA-OREL	3	2	00/01	M	U
256	8.51	-24.87	26.67	54.00	VOL-POST	1	1	00/01	M	U
262	9.54	-24.69	29.58	56.75	CHOLM	2	1	00/01	M	U
269	8.49	-24.47	26.42	53.00	STO-GON	3	2	00/01	F	U
270	7.05	-25.15	27.00	54.17	VOL-POST	1	1	00/01	M	U
276	9.77	-23.92	26.42	53.00	STO-GON	3	2	00/01	F	U
278	8.40	-24.58	26.42	53.00	STO-GON	3	2	00/01	M	U
280	10.32	-22.60	34.67	53.83	KA-OREL	3	2	00/01	M	U
281	10.21	-22.61	34.67	53.83	KA-OREL	3	2	00/01	F	U
287	10.63	-24.05	26.60	55.00	VOL-POST	1	1	00/01	M	U
288	10.20	-24.09	26.60	55.00	VOL-POST	1	1	00/01	F	U
291	11.16	-22.76	32.58	56.33	GAT	1	1	00/01	F	sad
306	9.73	-23.98	28.83	54.00	MIN	1	1	00/01	M	sad
326	11.93	-23.90	32.50	56.67	GAT	1	1	00/01	M	U
328	8.02	-25.80	30.50	56.83	CHOLM	2	1	00/01	F	ad
432	8.78	-25.07	34.25	55.58	SMO	2	1	01/02	M	U
434	10.69	-24.08	30.33	56.17	CHOLM	2	1	01/02	F	U
435	9.40	-23.72	30.33	56.17	CHOLM	2	1	01/02	F	U
436	11.41	-23.57	30.33	56.17	CHOLM	2	1	01/02	M	U
442	9.43	-23.19	35.67	53.42	KA-OREL	3	2	01/02	M	ad
445	8.27	-23.79	34.00	54.17	MED-UNE	1	2	01/02	M	ad
446	9.46	-24.73	30.50	58.00	CHOLM	2	1	01/02	M	ad
447	9.30	-24.66	30.50	58.00	CHOLM	2	1	01/02	M	sad

449	9.44	-23.85	35.17	53.25	KA-OREL	3	2	01/02	M	sad
452	9.88	-23.65	30.50	58.00	CHOLM	2	1	01/02	M	U
453	9.73	-22.41	27.50	51.65	STO-GON	3	2	01/02	M	U
454	9.47	-23.57	35.00	55.33	SMO	2	1	01/02	M	ad
456	8.49	-23.99	35.00	55.33	SMO	2	1	01/02	M	U
461	11.30	-23.63	31.50	55.67	SMO	2	1	01/02	M	U
463	8.19	-23.58	34.00	53.67	MED-UNE	1	2	01/02	M	U
464	9.47	-23.94	35.17	53.25	KA-OREL	3	2	01/02	F	U
491	9.42	-23.45	35.67	53.42	KA-OREL	3	2	01/02	M	sad
494	9.60	-23.33	27.50	51.65	STO-GON	3	2	01/02	F	U
495	10.44	-24.35	30.50	55.67	VIT	1	1	01/02	M	U
517	9.76	-23.33	29.00	57.33	LAT	1	1	02/03	F	U
518	9.06	-24.33	29.00	57.33	LAT	1	1	02/03	M	U
521	8.60	-24.53	29.00	57.33	LAT	1	1	02/03	F	U
522	8.69	-24.35	31.50	59.67	GAT	1	1	02/03	F	U
523	8.61	-24.06	31.50	59.67	GAT	1	1	02/03	M	U
524	7.99	-24.07	31.50	59.67	GAT	1	1	02/03	F	U
525	7.66	-24.64	31.50	59.67	GAT	1	1	02/03	F	U
565	11.35	-20.27	36.30	49.77	CHAR	4	2	01/02	M	U
776	8.26	-24.07	42.00	52.00	TAMB	3	2	03/04	M	U
777	8.55	-24.27	42.00	52.00	TAMB	3	2	03/04	F	U
778	8.78	-24.10	42.00	52.00	TAMB	3	2	03/04	M	U
779	8.87	-24.54	42.00	52.00	TAMB	3	2	03/04	F	U
780	9.15	-24.25	42.00	52.00	TAMB	3	2	03/04	F	U
781	8.34	-24.56	42.00	52.00	TAMB	3	2	03/04	M	U
