# Supplementary material for: Dietary Differentiation and the Evolution of Population Genetic Structure in a Highly Mobile Carnivore
Source: PLoS One. 2012 Jun 29;7(6):e39341. doi: 10.1371/journal.pone.0039341 (PMC3387138; doi:10.1371/journal.pone.0039341)
Supplement: Table S2 — Sample information and δ15N and δ13C isotope profiles (‰) for wolf prey species analyzed in this study. (PDF) [file pone.0039341.s004.pdf]

Table S2. Sample information and $\delta^{15}N$ and $\delta^{13}C$ isotope profiles (‰) for wolf prey species analysed in this study.

BIAL – Bialowieza Forest, Poland/Belarus
MIN – Minsk Area, Belarus
VIT – Vitebsk Area, Belarus
NE POLAND – north-eastern Poland
NE POLAND – north-eastern Poland
SE POLAND – north-eastern Poland
CE POLAND – central-eastern Poland

ID	Species	$\delta^{15}N$	$\delta^{13}C$	Region	Season
UNG1	Moose	2.62	-27.64	MIN	2007/2008
UNG3	Moose	3.33	-27.42	VIT	2007/2008
UNG13	Moose	6.37	-25.60	NE POLAND	2007/2008
UNG14	Moose	4.88	-27.51	NE POLAND	2007/2008
UNG18	Moose	5.25	-26.68	VIT	2007/2008
UNG4	Red deer	1.46	-28.62	NE POLAND	2004/2005
UNG5	Red deer	1.20	-26.87	NE POLAND	2004/2005
UNG9	Red deer	1.52	-26.82	NE POLAND	2004/2005
UNG10	Red deer	5.37	-25.00	BIAL	2005/2006
UNG11	Red deer	3.20	-27.72	BIAL	2004/2005
UNG6	Roe deer	3.52	-27.05	NE POLAND	2004/2005
UNG7	Roe deer	5.27	-24.54	NE POLAND	2004/2005
UNG8	Roe deer	4.66	-26.88	NE POLAND	2004/2005
UNG19	Roe deer	1.58	-26.93	NE POLAND	2005/2006
UNG20	Roe deer	3.42	-27.66	NE POLAND	2004/2005
UNG2	Wild boar	7.82	-20.84	NE POLAND	2004/2005
UNG12	Wild boar	6.46	-22.62	NE POLAND	2004/2005
UNG15	Wild boar	7.05	-16.00	NE POLAND	2004/2005
UNG16	Wild boar	4.91	-24.35	NE POLAND	2004/2005
Z001	European hare	1.06	-29.13	NE POLAND	2003/2004
Z003	European hare	0.91	-29.16	NE POLAND	2003/2004
Z401	European hare	5.90	-29.31	SE POLAND	2005/2006
Z402	European hare	6.75	-27.56	SE POLAND	2005/2006
Z403	European hare	8.18	-27.97	SE POLAND	2005/2006
Z535	European hare	6.60	-27.81	NE POLAND	2005/2006
Z801	European hare	8.41	-27.29	CE POLAND	2005/2006
Z803	European hare	7.59	-25.78	CE POLAND	2005/2006
Z806	European hare	6.89	-25.05	CE POLAND	2005/2006
B1	Beaver	2.93	-28.07	NE POLAND	2003/2004
B2	Beaver	6.87	-26.40	NE POLAND	2003/2004
B3	Beaver	6.89	-25.24	NE POLAND	2003/2004
B4	Beaver	7.03	-26.48	NE POLAND	2003/2004
B5	Beaver	7.53	-25.74	NE POLAND	2003/2004
