# Supplementary material for: Dietary Differentiation and the Evolution of Population Genetic Structure in a Highly Mobile Carnivore
Source: PLoS One. 2012 Jun 29;7(6):e39341. doi: 10.1371/journal.pone.0039341 (PMC3387138; doi:10.1371/journal.pone.0039341)
Supplement: Table S3 — Diet composition of wolves inferred from the stable isotope data using MixSIR. For: (A) four subpopulations delimited based on mtDNA variability (MIT 1- MIT 4), (B) two subpopulations delimited based on microsatellite variability (NUC 1, NUC 2), and (C) all individuals pulled together. The result for the subpopulation MIT 4 is based on one individual only and therefore is biased. We report median and 25th–75th percentile (25 and 75%ile) ranges. Because in some cases the posterior distributions are multimodal, these percentiles may not adequately describe the posterior surface of the source contributions. The posterior distributions are presented in the Figure S1. (PDF) [file pone.0039341.s005.pdf]

Table S3. Diet composition of wolves inferred from the stable isotope data using MixSIR for: (A) four subpopulations delimited based on mtDNA variability (MIT 1-MIT 4), (B) two subpopulations delimited based on microsatellite variability (NUC 1, NUC 2), and (C) all individuals pulled together. The result for the subpopulation MIT 4 is based on one individual only and therefore is biased. We report median and 25th–75th percentile (25 and 75%ile) ranges. Because in some cases the posterior distributions are multimodal, these percentiles may not adequately describe the posterior surface of the source contributions. The posterior distributions are presented in the Figure S1.

Each run leading to these results was performed twice, and the results were highly consistent. In each case, the number of posterior draws was higher than 20,000, there were no duplicate draws, each parameter vector sampled was unique, and the maximum importance ratio was below 0.0003, each of these parameters indicating good performance of the model. Additionally, the distribution of the posterior probabilities demonstrated that the model placed appropriate weight on the tails of the posterior distribution.

		Moose	Red deer	Roe deer	Wild boar	Hare	Beaver
(A)							
MEDIAN	MIT 1	0.039	0.307	0.072	0.214	0.217	0.041
25 %ile		0.016	0.081	0.027	0.193	0.073	0.017
75 %ile		0.081	0.383	0.266	0.232	0.274	0.090
MEDIAN	MIT 2	0.128	0.117	0.132	0.183	0.129	0.177
25 %ile		0.053	0.053	0.058	0.160	0.048	0.083
75 %ile		0.276	0.198	0.244	0.206	0.223	0.304
MEDIAN	MIT 3	0.143	0.204	0.240	0.285	0.034	0.077
25 %ile		0.087	0.159	0.176	0.270	0.016	0.043
75 %ile		0.203	0.248	0.302	0.300	0.059	0.116
MEDIAN	MIT 4	0.065	0.052	0.061	0.545	0.077	0.090
25 %ile		0.028	0.022	0.026	0.473	0.033	0.039
75 %ile		0.124	0.099	0.115	0.621	0.144	0.168
(B)							
MEDIAN	NUC 1	0.059	0.202	0.085	0.213	0.264	0.039
25 %ile		0.023	0.069	0.033	0.197	0.078	0.017
75 %ile		0.155	0.298	0.206	0.228	0.304	0.079
MEDIAN	NUC 2	0.107	0.240	0.237	0.299	0.032	0.057
25 %ile		0.055	0.182	0.154	0.284	0.014	0.028
75 %ile		0.172	0.293	0.322	0.314	0.058	0.094
(C)							
MEDIAN	ALL	0.042	0.275	0.097	0.249	0.090	0.068
25 %ile		0.018	0.047	0.033	0.232	0.027	0.025
75 %ile		0.086	0.371	0.513	0.266	0.184	0.153
