# Supplementary material for: Dietary Differentiation and the Evolution of Population Genetic Structure in a Highly Mobile Carnivore
Source: PLoS One. 2012 Jun 29;7(6):e39341. doi: 10.1371/journal.pone.0039341 (PMC3387138; doi:10.1371/journal.pone.0039341)
Supplement: Table S4 — Effects of ecological factors and geographical distance on genetic differentiation of Eastern European wolves. The tests were performed in the same way as those presented in Table for, but additional explanatory variables were considered. For details see Table 4 legend and Methods section with the description of the tests. (PDF) [file pone.0039341.s006.pdf]

Table S4. Effects of ecological factors and geographical distance on genetic differentiation of Eastern European wolves. The tests were performed in the same way as those presented in Table for, but additional explanatory variables were considered. For details see Table 4 legend and Methods section with the description of the tests.

	Marginal tests			Sequential tests		
Variable set	pseudo-F	*P*	%var	pseudo-F	*P*	%var
(A) Individual-based test						
Coordinates	3.2	0.0001	5.9	3.2	0.0001	5.9
Stable isotope composition	2.5	0.0001	4.6	2.3	0.0002	10.0
Moose presence/absence	6.1	0.0001	5.6	3.9	0.0001	13.4
Vegetation types	1.5	0.09	1.5	2.0	0.015	15.2
(B) Population-based test						
Stable isotope composition	4.7	0.006	46.1	4.7	0.006	46.1
Coordinates	3.6	0.018	39.3	3.1	0.032	68.0
Moose presence/absence	5.5	0.0006	31.5	2.3	0.187	75.1
Vegetation types	1.4	0.286	10.3	-	-	-*

*The sequential test ended prematurely because the last variable did not increase the genetic variation explained.
